# Supplementary material for: Temporal motifs in patent opposition and collaboration networks
Source: Sci Rep. 2022 Feb 4;12:1917. doi: 10.1038/s41598-022-05217-8 (PMC8817030; doi:10.1038/s41598-022-05217-8)
Supplement: Supplementary file 1 — Supplementary Tables. [file 41598_2022_5217_MOESM1_ESM.pdf]

Supplementary Information

Motif	LS	DCLS	WTS	IS	TS
R	N/A	N/A	210.4	11.2	160.6
P	None	None	112.8	4.2	45.6
I	1744.7	2173.7	121.3	4.8	89.6
O	76.8	-7.0	57.2	5.6	62.4
C	206.5	375.8	13.6	2.9	0.4
W	387.0	330.4	51.4	2.4	21.0
R-R	N/A	N/A	123.4	13.5	86.7
R-P	21.9	None	59.1	9.5	48.4
P-P	152794.5	None	62.5	1.5	36.6
P-R	56.4	None	80.7	1.4	40.6
R-O	211.1	7.8	72.2	5.8	62.1
R-C	151.2	183.1	103.9	4.5	106.9
R-W	20.3	337.5	76.7	14.1	79.0
R-I	21.3	1163.4	76.6	11.2	68.8
O-O	550020.7	12.5	32.6	0.9	19.7
O-R	141647.4	9.0	81.6	1.6	50.7
O-W	612179.5	None	35.8	2.2	30.2
O-I	34991.8	38198.3	65.6	1.2	31.9
O-P	None	None	61.3	3.0	70.4
O-C	None	34398.1	39.2	0.7	40.2
P-C	None	None	81.5	7.8	64.0
P-O	469151.2	None	40.2	8.5	37.2
P-I	281227.4	None	66.5	3.1	53.5
P-W	70429.2	None	61.0	3.8	51.1
C-W	162.4	90.7	60.3	2.7	33.3
C-I	None	97178.5	85.3	1.8	43.6
C-O	None	None	57.3	4.0	36.9
C-R	129.1	348.7	66.9	7.1	60.9
C-C	17332.6	None	31.5	1.7	30.6
C-P	None	None	92.4	2.3	58.0
W-C	171.8	349.0	90.2	5.0	35.2
W-P	None	None	43.7	2.1	41.9
W-I	None	None	138.7	4.7	58.5
W-W	24289.8	None	28.6	3.2	44.3
W-R	148.2	69.3	102.4	6.4	58.6
W-O	205318.6	55658.6	102.9	8.4	68.6
I-I	296.5	779.9	80.8	4.4	56.2
I-W	144813.8	51198.6	74.8	3.6	51.7
I-C	None	None	96.6	2.3	37.5
I-P	48411.5	None	80.8	2.9	71.6
I-O	99830.2	23996.4	65.7	3.8	44.1
I-R	497.0	655.2	179.5	4.9	108.2

Supplementary Table 1. *Z scores for the count of 2-event and 3-event motifs. The first six rows represent the 2-event motifs. The remaining rows represent the 3-event motifs. R-P, for example, indicates that the first two events in the 3-event motif produces R (i.e., repetition) and that the last two events in the same motif produces P (i.e., ping-pong). "None" indicates that the motif does not appear in any randomized network generated by the null model. "N/A" indicates that we observe the same number of motifs in all randomized networks by construction. In both cases, the Z score is undefined since the standard deviation of the motif count for the randomized networks is zero.*

Motif	Count	Number of collaborations			
		0	1	2	3
R-R	737852	100.00%	0.00%	N/A	N/A
R-P	64424	100.00%	0.00%	N/A	N/A
P-P	60427	100.00%	0.00%	N/A	N/A
P-R	93364	100.00%	0.00%	N/A	N/A
R-O	3935135	91.69%	8.31%	0.00%	0.00%
R-C	560573	98.92%	1.08%	0.00%	0.00%
R-W	960945	98.16%	1.84%	0.00%	0.00%
R-I	3020113	99.67%	0.33%	0.00%	0.00%
O-R	3990765	91.90%	8.10%	0.00%	0.00%
C-R	938454	95.16%	4.84%	0.00%	0.00%
W-R	744834	99.73%	0.27%	0.00%	0.00%
I-R	3323585	99.34%	0.66%	0.00%	0.00%
O-O	3671334	91.15%	8.85%	0.00%	0.00%
C-W	470241	99.23%	0.77%	0.00%	0.00%
W-C	663737	96.78%	3.22%	0.00%	0.00%
I-I	2065313	99.43%	0.57%	0.00%	0.00%
P-C	370553	94.08%	5.92%	0.00%	0.00%
P-O	593435	92.42%	7.58%	0.00%	0.00%
P-I	177864	99.17%	0.83%	0.00%	0.00%
P-W	178792	97.85%	2.15%	0.00%	0.00%
O-P	413190	95.82%	4.18%	0.00%	0.00%
C-P	181289	97.87%	2.13%	0.00%	0.00%
W-P	378198	94.37%	5.63%	0.00%	0.00%
I-P	183710	98.29%	1.71%	0.00%	0.00%
O-W	371236	95.38%	4.62%	0.00%	0.00%
C-O	518287	91.32%	8.68%	0.00%	0.00%
W-I	177805	99.17%	0.83%	0.00%	0.00%
I-C	151299	97.93%	2.07%	0.00%	0.00%
C-C	38368	100.00%	0.00%	0.00%	0.00%
C-I	173843	99.99%	0.01%	0.00%	0.00%
I-O	189415	99.98%	0.02%	0.00%	0.00%
I-W	183177	99.94%	0.06%	0.00%	0.00%
O-I	179172	99.99%	0.01%	0.00%	0.00%
O-C	174728	99.99%	0.01%	0.00%	0.00%
W-W	38406	100.00%	0.00%	0.00%	0.00%
W-O	198357	99.99%	0.01%	0.00%	0.00%

Supplementary Table 2. *The number of patent collaborations observed in each 3-event opposition motif. The total count and the percentages are given.*
